# Cause of Cholera

**Published:** 1874-06

**Authors:** 


### CAUSE OF CHOLERA.

A CORRESPONDENT of the Boston *Globe* enlightens the public on the causes of cholera. If his alleged causes are the true causes, then the remedy is easy. First, he says, epidemic cholera is in the atmosphere, but breathing the infected air will not of itself produce the disease. Second, the atmosphere infects the water most exposed to it, and both breathing the infected air and drinking the infected water produce the cholera. He says: "The poison in the atmosphere with which the water is saturated is, in my opinion, what causes epidemic cholera;" and he alleges the same of yellow fever. "Hence," he reasons, that "Boston, New York, Philadelphia, Chicago and other cities, using water isolated from the atmosphere, have an immunity from epidemic cholera," but not from "sporadic causes of cholera," which arise in the careless use of water that has long been exposed to the cholera infected atmosphere. "If," he argues, "these cities were in the use of water from wells and cisterns, they would be scourged by cholera in an epidemic form," as they were in the days of 1832–33, before the era of water-works. Circumstances give plausibility to this theory. The Asiatic cholera this year has not taken an epidemic form in any of the large cities of the Old or New World; but it has been very fatal in a good many small towns in the West and in Europe. The small towns use water exposed to the poisoned air; the large cities are now nearly all supplied with water through pipes which exclude it from the infected air. There have been but a few cases of cholera in Cincinnati or St. Louis, none reported in Chicago, only a few at Louisville, and none in the great cities of New York, Brooklyn, Philadelphia, Baltimore, Boston and Pittsburg. Along the Ohio and Mississippi rivers, the small towns, where wells and cisterns supply the drinking water, the epidemic has taken on occasionally its most frightful form. We give the concluding part of the Boston paper's communication:

"If our cities were in the use of water from wells and cisterns, limestone or no limestone, they would be scourged with this disease, as they were in the days of our fathers, when it was looked upon as coming direct from the hand of '*Providence.*' In the year 1848 I promulgated this same theory, saying that the cities named, by the enterprise of bringing water into them, isolated from the atmosphere, had exempted themselves from the pestilential character of both cholera and yellow fever epidemically; and have not heard of any facts since to cause any change in my mind. If in those sections and places where they are not blessed, as our large cities named are, they would take the water from wells, &c., and boil it for half an hour, and put it in air-tight vessels and keep it in a cool place for the next day's use, cholera would not, in my opinion, put on an epidemic form, even in limestone sections of the country."

